# Stem Cell-derived Exosomal MicroRNA as Therapy for Vascular Age-related Diseases

**DOI:** 10.14336/AD.2021.1110

**Published:** 2022-06-01

**Authors:** Hang Ren, Ziyuan Guo, Yang Liu, Chunli Song

**Affiliations:** Department of Cardiovascular Internal Medicine, the Second Hospital of Jilin University, Changchun, China

**Keywords:** MicroRNAs, senescence, stem cell-derived exosomal microRNA, vascular age-related diseases

## Abstract

Vascular age-related diseases describe a group of age-related chronic diseases that result in a considerable healthcare burden to society. Vascular aging includes structural changes and dysfunctions of endothelial cells (ECs) and smooth muscle cells (SMCs) in blood vessels. Compared with conventional treatment for vascular age-related diseases, stem cell (SC) therapy elicits better anti-aging effects *via*the inhibition/delay ECs and SMCs from entering senescence. Exosomal noncoding RNA (ncRNAs) in vascular aging and stem cell-derived exosomal microRNAs (SCEV-miRNAs), especially in mesenchymal stem cells, have an important role in the development of age-related diseases. This review summarizes SCEV-miRNAs of diverse origins that may play a vital role in treating subclinical and clinical stages of vascular age-related disorders. We further explored possible age-related pathways and molecular targets of SCEV-miRNA, which are associated with dysfunctions of ECs and SMCs in the senescent stage. Moreover, the perspectives and difficulties of SCEV-miRNA clinical translation are discussed. This review aims to provide greater understanding of the biology of vascular aging and to identify critical therapeutic targets for SCEV-miRNAs. Though still in its infancy, the potential value of SCEV-miRNAs for vascular age-related diseases is clear.

## 1. Introduction

**Figure 1.**
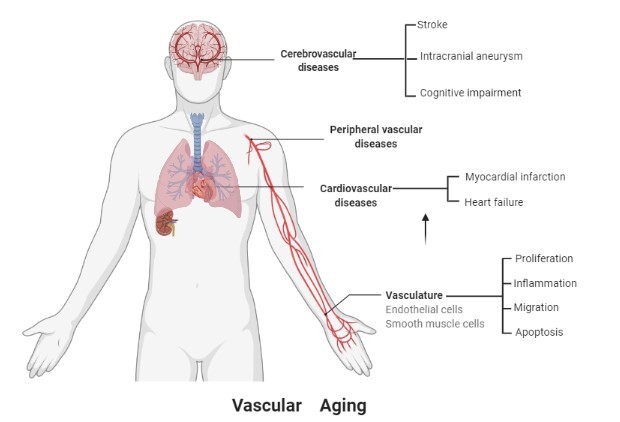
Vascular aging mainly includes age-related to cardiovascular, cerebrovascular, and peripheral vascular diseases. Vascular ageing may be caused by the dysfunction of endothelial and smooth muscle cells (SMCs), which includes abnormal proliferation, inflammation, migration, apoptosis, and angiogenesis.

**Figure 2.**
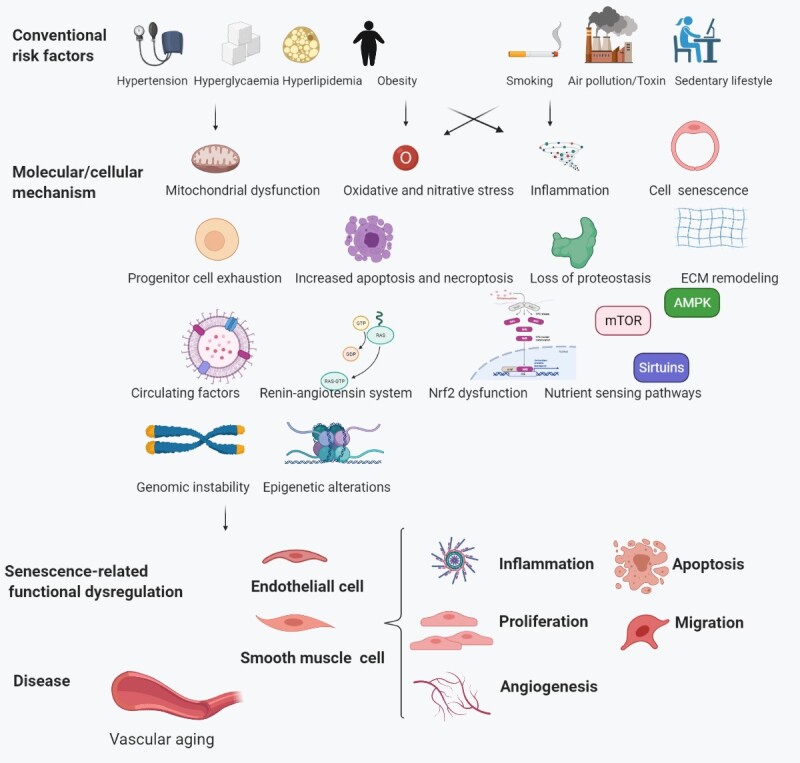
Risk factors associated with aging. Traditional risk factors activate the cellular and molecular mechanisms of vascular aging, resulting in age-related dysfunction of endothelial and smooth muscle cells (SMCs), and ultimately, vascular aging diseases.

There is increasing evidence that most major vascular diseases are interconnected with the biology of aging [1]. Vascular age-related disorders mainly affect the cardiovascular, cerebrovascular, and peripheral vascular systems, causing coronary artery disease (CAD), hypertension, and stroke (Fig. 1). However, the specific mechanisms that are involved in vascular aging are multifactorial and unclear. Treatments for vascular age-related diseases, including pharmacotherapy, structured exercise, and lifestyle modification, have been widely used to treat vascular-age related diseases. However, adverse side effects have been reported during pharmacotherapy. For example, rapamycin may cause metabolic disorders, and metformin may cause gastrointestinal disorders [2]. Given the increasing aging population, it is crucial to understand the molecular mechanisms underlying vascular aging and explore more efficient therapeutic strategies (Fig. 2). Although still in its initial stage, SCEV-miRNA therapy in vascular age-related diseases has shown significant potential therapeutic and clinical application. We briefly summarized the current studies regarding the therapeutic role of SCEV-miRNAs in vascular age-related diseases and clarified their possible age-dependent molecular or pathways downstream.

## 2. Aging and health

Aging is a significant healthcare challenge for China. By 2050, an estimated 400 million Chinese citizens will be aged > 65 years, 150 million of whom will be > 80 years [3]. Aging is the primary driver for most chronic diseases [4]. As vascular aging is a specific type of organic aging, new therapeutic approaches for vascular aging are urgently needed. For instance, anti-aging molecules, such as nicotinamide adenine dinucleotide (NAD+), may be promising therapeutic targets as interventional strategies [4, 5]. Vascular aging can be reversed by the impairment of the endothelial NAD+-H_2_S signaling network [6]. Using NAD+ augmentation, the accelerated aging in Werner syndrome, a classical premature aging disease, is limited [7].

## 3. EC and SMC senescence

As endothelial cells (EC) and smooth muscle cells (SMC) are the main components of the inner and middle layers of the vascular wall, respectively, their structural and functional dysfunctions are the leading causes of vascular aging, primarily characterized by age-related phenotypes, such as abnormal proliferation, inflammation, migration, angiogenesis, and apoptosis [8]. Healthy ECs maintain a balance between vascular vasodilation and contraction, proinflammatory and anti-inflammatory activities, as well as thrombotic and antithrombotic activities. With vascular aging, ECs lose this ability [9]. Healthy SMCs display a contractile phenotype that regulates the blood vessel’s structure and function. Vascular aging induces phenotypic alterations in SMCs, which function in arterial calcification and atherosclerosis [10].

Senescent vascular cells communicate with their neighbors via direct cell-to-cell contact and indirect secretory factor-dependent signaling and the altered senescence-associated secretory phenotype (SASP). These events diminish stem cell (SC) regeneration and accelerate vascular aging [11, 12]. Biological effects accompanying senescence-associated risk factors, such as hypertension, hyperglycemia, hyperlipidemia, obesity, smoking, environmental pollutants or toxin exposure, and sedentary lifestyles, have become more pronounced, resulting in EC and SMC senescence and further causing vascular aging diseases (Fig. 2).

## 4. SCEV-miRNA in vascular aging

Stem cells are broadly defined as self-renewing precursor cells with the ability to differentiate into various cell types. SC depletion is an essential mechanism of vascular aging [13]. As cell therapy, SC supplementation can enhance the stemness of dormant cells and their anti-senescence properties [14], delaying or even reversing aging *in vivo* [15]. However, some issues, such as low survival rates after transplantation, tumorigenicity, immunogenicity, low tissue-targeting effects, and ethical concerns limit their clinical applications [16-19].

Extracellular vesicles (EVs) are lipid bilayer-containing particles released from cells, both in physiological and pathological conditions [20]. Stem cell-derived extracellular vesicles (SCEVs) retain the anti-aging characteristics [21] and avoid tumorigenesis and immune rejection of SCs [22]. As therapeutic gene vectors, SCEVs are less immunogenic than nanoparticles and less toxic compared with adenoviruses. Additionally, they can cross the blood-brain barrier (BBB) and other biological barriers and are easier to store and transport than other gene vectors [22-24].

As a crucial functional cargo of EVs, microRNAs (miRNAs) are small noncoding RNA molecules of approximately 22 nucleotides [25]. They mainly engage the 3′- untranslated region (UTR) of the target mRNA followed by the initiation of target degradation or inhibition of translation. They participate in the epigenetic regulation of vascular age-related dysfunctions. The majority of senescence-inducing signals engage either or both of the p53/p21 and p16/pRb pathways as the final effectors of the cellular senescence program [26]. Most senescence-regulatory miRNAs, such as micro34a and micro106a, have been further confirmed to regulate p53/p21 and p16/pRb pathways signaling pathways either directly or indirectly. However, there is still little information regarding the potential involvement of miRNAs in modulating the complex process of cellular senescence. Some other miRNAs have a demonstrated link to the complex senescence regulatory networks, causing further vascular aging phenotypes, but with no significant evidence on their relevance to the two aforementioned classic pathways [26].

**Table 1:** Identified senescence-related SCEV-miRNA, their possible direct pathways/targets downstream and altered senescence-related cellular phenotypes.

SCEV-miRNAs	Possible direct pathways/ targets	Altered senescence-related cellular phenotypes					Ref.
		Proliferation	Migration	Apoptosis	Angiogenesis	Inflammation	
miR-199b-5p	Jagger-1/Notch1 pathway	√	√		√		[1]
miR-21, miR-21-5p, miR-221-3p	PTEN/AKT pathway	√	√	√	√		[2-4]
miR-218	Ribo-1		√				[5]
miR-181b-5p	TRPM7		√		√		[6]
miR-512-3p	Keap1	√		√		√	[7]
miR-145	JAM-A		√				[8]
miR-342-5p	PPP1R12B			√			[9]
miR-210	Efna3	√	√		√		[10]
miR-132	RasGAP-p120				√		[11]
miR-210	nSMase2			√	√		[12]
miR-204, miR-17 superfamily	STAT3 pathway				√	√	[13]
miR-292, miR-103, miR-17, miR-210	Profibrotic gene				√		[14]
miR-125a	DLL4				√		[15]
miR-21-5p	THBS1	√	√		√		[16]
miR-126	SPRED1					√	[17]
miR-132-3p	RASA1			√			[18]

SCEV-miRNA, a combination of gene and cell therapy, is associated with the senescence process. For example, aging speed is controlled partly through hypothalamic stem cell-derived exosomal miRNAs. Mechanistically, hypothalamic stem/progenitor cells contribute significantly to exosomal miRNAs in the cerebrospinal fluid, and the level of these exosomal miRNAs declines during aging. In contrast, treatment with healthy hypothalamic stem/progenitor cell-secreted exosomes slows aging [27]. Exogenous therapy comprising SCEV-miRNAs may regulate age-related dysfunctions and further cure vascular age-related diseases through the pathways/targets mentioned above. We summarized some recently identified SCEV-miRNAs related to vascular cellular senescence, their pathways and targets downstream, and altered senescence phenotypes of EC and SMC in Table 1.

**Figure 3.**
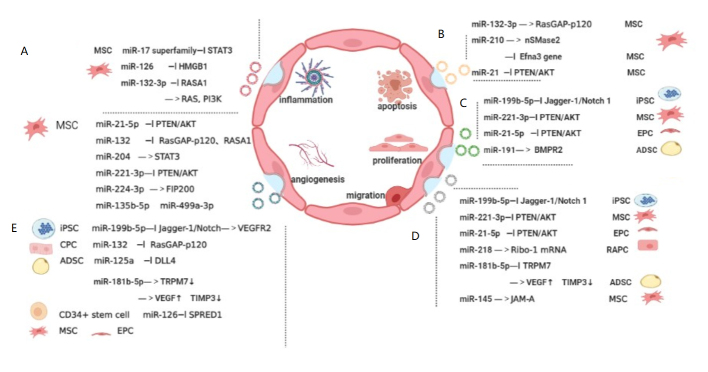
Role of SCEV-miRNAs in EC senescence. Extracellular microRNAs derived from stem cells (SCEV-miRNAs) act on endothelial cells (ECs), by regulating mRNA expression of genes involved in senescence-related dysfunctions, including abnormal inflammation, apoptosis, proliferation, migration, and angiogenesis.

**Figure 4.**
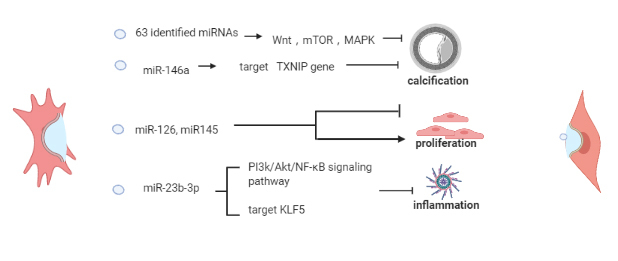
Role of SCEV-miRNAs in SMC senescence. Extracellular microRNAs derived from stem cells (SCEV-miRNAs) act on smooth muscle cells (SMCs), regulating senescence-related cellular phenotypes, including abnormal inflammation, apoptosis, proliferation, migration, and angiogenesis.

Notably, the possible therapeutic modulatory effect of SCEV-miRNA on EC angiogenesis is also essential for wound healing, tissue repair, and cancer development, which do not belong to vascular age-related diseases. Related research that does not mention specific vascular age-related diseases was excluded for clarity [28, 29]. Considering its potent anti-aging effects and prospects, SCEV-miRNA therapy for vascular age-related diseases, which targets ECs and SMCs [30, 31] (Fig. 3 and Fig. 4). SCEV-miRNAs regulate the mRNA expression that are involved in senescence-related dysfunctions including abnormal inflammation, apoptosis, proliferation, migration, and angiogenesis. The mesenchymal stem cell (MSC)-derived exosomal miR-17 superfamily, which include miR-126, and miR-132-3p, regulate inflammation-related mRNAs, such as signal transducer and activator of transcription 3(STAT3), high-mobility group box 1 protein (HMGB1), RAS, and PI3K, respectively [32-34] (Fig. 3A). The MSC-derived exosomal miR-132-3p, miR-210, and miR-21, regulate apoptosis-related mRNAs, which include RasGAP-120 [35], neutral type II sphingomyelinase (nSMase2) [36], and phosphatase and tensin homolog/protein kinase B (PTEN/AKT) [37], respectively (Fig. 3B). The induced pluripotent stem cell (iPSC)-derived exosomal miR-199-5p regulate cell proliferation, migration, and angiogenesis by targeting the Jagger-1/Notch 1 pathway [38]. MSC-derived exosomal miR-221-3p and endothelial progenitor cell (EPC)-derived exosomal miR-21-5p regulate the proliferation, migration, and angiogenesis of ECs by targeting PTEN/AKT [39, 40]. Adipose tissue-derived stem cell (ADSC)-derived exosomal miR-191 regulates proliferation-related BMPR2 [41] (Fig. 3C). The renal artery-derived vascular progenitor cell (RAPC)-derived exosomal miR-218, ADSC-derived exosomal miR-181b-5p, and MSC-derived exosomal miR-145 regulate migration-related mRNAs, Ribo-1 [42], vascular endothelial growth factor (VEGF) [43], and junction adhesion molecule A (JAM-A )[44] respectively (Fig. 3D). The MSC- and cardiac progenitor cell (CPC)-derived exosomal miR-132 regulates angiogenesis-related RasGAP-120 [35]. MSC-derived exosomal miR-204 regulates angiogenesis-related STAT3 [32]. ADSC-derived exosomal miR-125a and miR-181b-5p regulate delta-like 4(DLL4) [28] and VEGF [43], respectively. The MSC, EPC, and CD34+ stem cell-derived exosomal miR-126 regulate angiogenesis-related sprouty-related EVH1 domain-containing protein 1(SPRED1) [33] (Fig. 3E). Meanwhile, SCEV-miRNAs also act on SMCs by regulating the mRNA expression of genes that participate in senescence-related dysfunctions. The senescence-related calcification response of SMCs can be altered by 63 specific miRNAs [45] and miR-146a of MSC-derived exosomes (MSC-EXOs) by targeting the *TXNIP*[46]. The senescence-related inflammation response can be altered by the miR-23b-3p in MSC-EXOs [47]. The senescence-related proliferation response can be altered by the miR-126 and miR-145 in MSC-EXOs [48] (Fig. 4).

## 5. SCEV-miRNAs as therapy for vascular age-related diseases

The course of vascular age-related diseases can be divided into subclinical and clinical stages. The subclinical stage mainly refers to age-related pathological changes, such as vascular calcification and atherosclerosis. The clinical setting mainly pertains to age-related cardiovascular, cerebrovascular, and peripheral vascular diseases. Next, we summarized the current progress in SCEV-miRNA therapy at the subclinical and clinical stages of vascular age-related disorders from the perspective of clinical applications.

### 5.1 SCEV-miRNAs and the subclinical stage of vascular age-related diseases

Vascular calcification refers to the deposition of ectopic calcium salts in blood vessels caused by abnormal calcium and phosphorus metabolism. It is an essential pathological phenotype associated with vascular aging. The cytological basis of vascular calcification originates from the differentiation of vascular SMCs (VSMCs) into cells with an osteoblast-like phenotype and is regulated by various miRNAs [49]. Mounting evidence suggests that SCEV-miRNAs may pose an anti-calcification function (Table 2). During osteogenic differentiation, exosomal miRNAs isolated from MSCs were profiled using miRNA arrays. Among 79 miRNAs detected, nine exosomal miRNAs were upregulated, and four miRNAs were downregulated. Five miRNAs (miR-199b, miR-218, miR-148a, miR-135b, and miR-221) were further validated and differentially expressed at different time points indicating their potential anti-aging therapeutic effects [50]. Beyond that, miR-146a, an overexpressed replicative senescent EC, is involved in important cell functions (growth, proliferation, death, survival, and maintenance) and age-related diseases (cancer, skeletal and muscle disorders, neurological, cardiovascular, and metabolic diseases). MiR-146 participates in the regulation of vascular aging partly through modulating mitochondrial integrity and function in aging cells, and by inducing or contributing to the inflammatory response and age-related diseases [31]. MiR-146a expression in MSC-derived exosomes (MSC-EXOs) significantly increases after stimulation with advanced glycation end products, which further attenuates calcification by targeting thioredoxin-interacting protein (TXNIP). TXNIP regulates cellular senescence by inhibiting the AKT pathways via direct interactions under conditions of metabolic stress [46, 51]. After stimulation with high phosphorus levels, 63 exosomal miRNAs derived from bone marrow-derived mesenchymal stem cells (BMSCs) were found to be significantly upregulated, which inhibited high phosphorus-induced calcification in human aortic VSMCs. MiRNAs activate the mechanistic target of rapamycin (mTOR), mitogen-activated protein kinase (MAPK), and Wnt signaling pathways, which are regulatory pathways of aging, and thus alleviate age-related vascular calcification [2, 45]. Research regarding SCEVs is progressing towards the pre-clinical stage with increasing attention focused on MSC-derived EVs [48, 52]. For example, vascular grafts often exhibit low patency rates within patients after surgery. When a graft functionalized by MSC-derived EVs was applied to hyperlipidemic rats, the results showed that incorporating MSC-derived EVs inhibited vascular thrombosis and calcification, enhancing graft patency. Furthermore, miR-126 and miR-145 were shown to be enriched in MSC-EXOs [48, 52]. The age-related anti-calcification effect of MSC-EXOs is attributable to the presence of these miRNAs, which inhibit endothelial dysfunction and regulate cellular replicative senescence. Related studies provide a necessary theoretical basis for the clinical applications of these findings to coronary artery bypass grafting, thrombosis, and vascular calcification [48].

Atherosclerosis is a vital pathology that is associated with vascular aging and characterized by intimal plaques and cholesterol accumulation in the arterial wall. These changes result in arterial wall stiffness and stenosis. We found that the transfer of miRNA-221 from MSC-EVs inhibits atherosclerotic plaque formation [53]. Atherosclerotic plaques contain aged ECs and SMCs [54, 55]. Additionally, senescent cells accumulate during aging and contribute to the pathogenesis of atherosclerosis, as well as other age-related disorders and chronic diseases. Apolipoprotein E-deficient mice fed a high-fat diet (HFD) also presented signs of senescent ECs in their blood vessels, eventually leading to atherosclerosis [56]. The removal of senescent cells via “senolysis” has been reported to reduce atherogenesis [57]. Vascular cellular dysfunction contributes to the alterations in extracellular matrix (ECM) proteins, resulting in vascular stiffness and loss of elasticity, and the proinflammatory state promotes atherosclerosis. The possibility of preventing accelerated cellular senescence is a therapeutic target in atherosclerosis [55]. SCEV-miRNAs can be used to treat age-related atherosclerosis (Table 2). For example, miR-342-5p, differentially expressed in healthy individuals and patients with atherosclerosis, promotes EC apoptosis via mitochondrial-related pathways [58]. *PPP1R12B*, a target gene of miR-342-5p related to VSMC contraction and oxytocin signaling, promotes the occurrence and development of atherosclerosis. In a model of atherosclerosis, reduced expression of miR-342-5p in ADSC-derived EV targets *PPP1R12B*to combat aging-associated atherosclerosis [58]. In addition, SCEV-miRNAs inhibit the formation or reduce the size of plaques. For example, increased levels of miR-145 in mesenchymal SC-derived extracellular vesicles (MSC-EVs) inhibit the formation or rupture of plaques in CAD patients by downregulating JAM-A [44]. MiR-512-3p, enriched in MSC-EXOs, markedly inhibits ox-LDL-mediated EC dysfunction, accelerates cell proliferation, inhibits apoptosis, and suppresses the levels of inflammatory cytokines by targeting Kelch-like ECH-associated protein 1 (Keap1) [59].

**Table 2:** SCEV-miRNAs associated with the subclinical stage of vascular aging diseases.

Process	Stem cells	Recipient cells	Exosomal cargo	Targeted or pathways	Senescence-related cell functions	Ref.
Calcification	MSCs	VSMCs	63 miRNAs identified	Wnt, mTOR, MAPK	Anti-calcification	[19]
Calcification	MSCs	VSMCs	miR-146a	3′ UTR of TXNIP gene	Anti-calcification	[20]
Calcification	MSCs	ECs/VSMCs	miR-126 miR-145	——	Anti-calcification, promotes proliferation	[21]
AS	MSCs	ECs	miR-145	Downregulates JAM-A	Anti-migration	[8]
AS	ADSCs	ECs	miR-342-5p	*PPP1R12B*	Anti-apoptosis	[9]
AS	MSCs	ECs	miR-512-3p	regulating Keap1	Promotes proliferation, anti-apoptosis Anti-inflammation	[7]

### 5.2 SCEV-miRNAs and the clinical stage of vascular age-related diseases

#### 5.2.1 SCEV-miRNAs and cardiovascular age-related diseases

Vascular aging, a retrogressive change, is a leading cause of cardiovascular diseases [60]. SCEV-miRNAs inhibit fibrosis and promote angiogenesis of infarcted regions by regulating cellular dysfunction of vascular aging. The therapeutic effect depends on the type of SC, tissue of origin, donor age, and cell microenvironment (Table 3). MiRNAs, protein cargo, and biological effects of EVs vary by SC type. For example, MSC-EVs contain high amounts of miR-23b, miR-124a, miR-126, and miR-221, promote angiogenesis, and inhibit apoptosis and fibrosis in vascular aging. In addition, MSC-EVs exert antitumor, anti-insulin resistance, and immunoregulatory effects. Furthermore, as donor cells for heart transplantation, CPCs can improve cardiac function and promote cardiovascular formation through CPC-EVs [61]. The elevated expression of miR-132 in CPC-EVs after myocardial infarction is related to cellular senescence and stemness [62]. The miR-132-induced downregulation of RasGAP-p120 improves collateral circulation in the infarcted area by promoting angiogenesis [35, 63]. Recent study has confirmed miR-132 as a target for heart failure (HF) therapy, with anti-miR-132 treatments having considerable clinical potential [64]. Differentially expressed during aging, miR-103 and miR-17 might induce the reverse regulation of aging-associated pathways [65]. MiR-103-3p is associated with the regulation of reduced cardiac apoptosis and promotes cardiac repair through the GSK3β/β-catenin pathway in acute myocardial infarction (AMI) [66], while miR-17-5p-mediated endoplasmic reticulum stress promotes AMI [67]. MiR-210 expression is also altered in age-related cardiovascular diseases such as atherosclerosis, acute myocardial infarction, and heart failure [68]. Circulating miR-210 concentrations are increased in patients with HF [69]. The levels of miR-103, miR-17, and miR-210 are elevated in CPC-EVs under hypoxic conditions, promoting angiogenesis and inhibiting fibrosis [70]. Finally, cardiosphere-derived cells are also the donor cells for cardiovascular age-related diseases [71]. Donor cells impact the therapeutic effect of SCEV-miRNAs. MSCs derived from the endometrium exert a more substantial paracrine influence and better angiogenic effect than BMSCs and ADSCs. In an AMI rat model, increased expression of miR-21 in endometrium-derived mesenchymal stem cell (EnMSC)-EVs improved clinical prognosis by inhibiting the PTEN/AKT pathway and EC apoptosis and by promoting angiogenesis [37]. However, downregulated miRNA-21 in MSC-EVs of HF patients increases proliferation and migration and suppresses angiogenesis of ECs [40].

**Table 3:** SCEV-miRNAs associated with aging-related cardiovascular diseases.

Process	Stem cells	Recipient cells	Exosomal cargo	Targets or pathways	Senescence-related cell function	Ref.
AMI	EnMSCs	ECs	miR-21	PTEN/AKT	Anti-apoptosis, pro-angiogenesis	[2]
AMI	MSCs (Young donors, 20-25 years old)	ECs	miR-221-3p	PTEN/AKT	Pro-angiogenesis, anti-fibrosis, pro-proliferation, pro-migration	[3]
AMI	BM-MSCs	ECs	miR-210	Increase the expression of nSMase2 under hypoxic conditions	Anti-apoptosis, pro-angiogenesis	[12]
AMI	BM-MSCs	ECs	miR-210	Inhibits the expression of *Efna3* gene	Pro-angiogenesis, pro-proliferation, pro-migration	[10]
AMI	CDC	ECs	miR-12, miR-130a, miR-210	—	Pro-angiogenesis	[22]
AMI	CPC	ECs	miR-132	Downregulates RasGAP-p120	Pro-angiogenesis	[11]
AMI	CPC	ECs	miR-292, miR-210, miR-103, miR-17	Decreases profibrotic gene expression	Pro-angiogenesis, anti-fibrosis	[14]
HF	Cardiac stromal cells	ECs	miR-21-5p	PTEN/AKT	Pro-proliferation, pro-migration	[4]
HF	BM-MSCs	ECs	23 miRNAs identified	—	Anti-apoptosis, pro-angiogenesis	[23]

The regenerative activity of SCEVs from young donors is more pronounced than that of cells from old donors. Neonatal umbilical cord-produced MSC-EVs contain abundant anti-aging signals and rejuvenate senescent adult BMSCs [72]. For example, miR-221-3p, an anti-aging indicator highly expressed in age-related diseases, is abundant in MCS-EVs from young donors. MiR-221 functions in telomere maintenance and regulates the cell cycle [73] by reducing fibrosis and inhibiting the PTEN/AKT pathway and promoting proliferation, migration, and angiogenesis of ECs [39]. Hence, young donors have high translational value in anti-aging intervention as prominent EV donors for vascular age-related diseases.

The therapeutic effects of SCEV-miRNAs depend on their microenvironment. For example, the paracrine effect of MSCs is potentiated by ischemia-hypoxia. EV biogenesis is regulated by nSMase2 and Rab proteins. nSMase2 expression increases under ischemia-hypoxia, causing more pronounced paracrine and therapeutic effects from SCEV-miRNA. Injecting C57BL/6 AMI mice with MSC-EVs cultured under hypoxic or normoxic conditions revealed that miR-210 expression is elevated in mice that received hypoxic MSC-EVs [36]. MiR-210 is upregulated by hypoxia-inducible factor 1α (HIF1-α) under hypoxic conditions, which results in cell cycle arrest and senescence via inhibition of the E2F transcription factor family [74]. MiR-210 induces chromosomal aberrations, double-stranded DNA breakage, and reactive oxygen species (ROS) accumulation, thereby promoting a senescent phenotype [75]. In cardiovascular age-related diseases, miR-210 promotes angiogenesis and inhibits apoptosis in ECs. Therefore, hypoxic preconditioning of donor cells can optimize the therapeutic effects of SCEV-miRNAs [36]. Intramyocardial injection of MSC-EVs into the left anterior descending coronary artery-infarct zone in murine infarction models resulted in a selective elevation of miR-210 levels, which improved cardiac functions after myocardial infarction [76]. In addition, bioactive and chemical pretreatment of donor cells impacts the therapeutic effect of miRNAs.

A network analysis of miRNA-profiled MSC-EXOs was performed to identify the dominant biological processes and pathways modulated by exosomal miRNAs. An analysis of crucial landscape pathways modulated by exosomal miRNAs revealed a significant increase in the levels of 23 specific miRNAs enriched in cardiovascular and angiogenesis processes [77]. By acting on more than 5,000 downstream genes, miRNAs promote angiogenesis and inhibit apoptosis. The anti-vascular aging effect is achieved by the targeted regulation of related miRNAs.

**Table 4:** SCEV-miRNAs associated with aging-related cerebrovascular diseases.

Process	Stem cells	Recipient cells	Exosomal cargo	Targets or pathways	Senescence-related cell functions	Ref.
Ischemic stroke	ADSCs	ECs	miR-181b-5p	Upregulates HIF-1α and VEGF, downregulates TRPM7 and TIMP3	Pro-migration, angiogenesis	[6]
Ischemic cerebrovascular disease	MSCs	ECs	miR-132-3p	Upregulates RAS and PI3K, downregulates RASA1	Anti-apoptosis	[18]
Brain ischemic injury	EPCs	ECs	miR-21-5p	Inhibits THBS1	Pro-proliferation, migration, vascular repair	[16]
Vascular disease of spinal cord	MSCs	ECs	miR-126	Inhibits SPRED1 and PIK3R2 expression	Pro-angiogenesis	[17]
Intracranial aneurysm	BM-MSCs	VSMCs	miR-23b-3p	Suppresses the PI3k/AKT/NF-κB signaling pathway, targets KLF5	Anti-inflammatory, improves pathological remodeling of the intracranial aneurysm wall	[24]

#### 5.2.2 SCEV-miRNAs and cerebrovascular age-related diseases

The term cerebrovascular disease refers to various conditions, including cerebral atherosclerosis, stenosis, occlusion, cerebral artery injury, and cerebral aneurysm. The brain consumes the most oxygen in the human body, and its structure and function are heavily dependent on the blood supply. Vascular aging makes the brain prone to ischemic and hemorrhagic injury with secondary brain atrophy, BBB damage, sensorimotor function, and cognitive decline. Age-related cerebrovascular diseases include stroke, cognitive aging, vascular cognitive impairment, dementia [78], Alzheimer's disease [79], and cerebral aneurysm (Table 4). ECs are an essential component of the neurovascular unit, comprising pericytes, ECs in cerebral vessels, parenchymal brain cells, and intercellular junctions of the BBB [80]. Endothelial aging affects vascular homeostasis, remodeling, and BBB integrity; endothelial dysfunction plays a crucial role in vascular aging [81].

SCEV-miRNAs play a role in age-related cerebrovascular diseases by modulating senescence-related miRNAs. As an essential regulator of endothelial cellular senescence and nerve injury, miRNA expression varies with age. For example, levels of age-related miRNAs, such as miR-34a and miR-29, are altered after stroke. SCEV-miRNA promotes EC proliferation and migration to form a vascular scaffold, inhibiting EC apoptosis from reducing the injury area size. For example, miR-181b expression is elevated in the ECs of the elderly, which is associated with endothelial cell senescence and the regulation of neovascularization [82]. Furthermore, miR-181b-5p is upregulated in ADSC-EVs after stroke [43]. MiR-181b-5p enhances axonal growth and suppresses apoptosis to reduce the infarction area by targeting the suppressor of TRPM7; however, it also upregulates the expression of VEGF, promoting angiogenesis and neurogenesis [43]. Another miRNA, miR-132, is a marker of vascular aging. After cerebral ischemia, miR-132-3p overexpression in MSC-EVs downregulates target protein RASA1 levels. It upregulates the expression of Ras and downstream PI3K phosphorylation, which improves vascular age-related ischemic injury by inhibiting cellular apoptosis [34]. As another example, in a rat model of carotid artery injury, age-related miR-21-5p expression is increased. Overexpression of miR-21-5p promotes age-related dysfunction by inhibiting thrombospondin 1 (THBS1). THBS1 causes vascular dysfunction and ischemia by inducing reactive oxygen species (ROS) production and disrupting vasodilation in the aging coronary artery [83, 84]. Furthermore, miR-126 levels in ECs are related to the stage of aging [85], and the expression of MSC-EV miR-126 increases after spinal cord ischemic injury [33]. By promoting the expression of SPRED1 and phosphoinositide-3-kinase regulatory subunit 2 (PIK3R2), miR-21-5p promotes angiogenesis and nerve regeneration, which improves vascular age-related dysfunction after spinal cord injury in the rat. Finally, the expression of miR-23 downregulates lamin-B1 (LMNB1), which is an essential regulator of aging. Increased expression of miR-23b-3p in MSC-EVs inhibits the inflammatory response. Moreover, it improves age-related remodeling of the intracranial aneurysm wall by inhibiting the PI3K/AKT/NF-κB signaling pathway in SMCs, relieving intracranial aneurysms [47].

**Table 5:** SCEV-miRNAs associated with aging-related peripheral vascular diseases.

Process	Stem cells	Recipient cells	Exosomal cargo	Targets or pathways	Senescence-related cell functions	Ref.
PAH	ADSCs	ECs	miR-191	Regulates BMPR2	Pro-proliferation	[25]
PAH	MSCs	ECs	miR-204	STAT3	Pro- angiogenesis	[13]
PAH	MSCs	ECs	miR-17 superfamily	Inhibits STAT3	Anti-inflammation	[13]
PAD	iPSCs	ECs	miR-199b-5p	Inhibits the Jagged-1/Notch1 signaling, pathway upregulates VEGFR2	Pro-proliferation, migration, angiogenesis	[1]
PAD	iVPC	ECs	miR-143-3p, miR-291b, miR-20b-5p	Pentraxin-3 and insulin-like growth factor-binding protein-3	Pro-angiogenesis	[26]
PAD	Cardiac MSC	Ischemic limb	miR-7116-5p	Regulates protein ubiquitination	Pro-proliferation	[27]
PAD	Stem cell	H2C9	miR-675		Anti-senescence	[28]
Renal injury	RAPC	ECs	miR-218	Targets *Robo1*	Pro- migration	[5]
Erectile dysfunction	ADSCs ADSCs	ECs ECs	miR-126 miR-130a miR-132 miR-let7b miR-let7c	——	Pro-angiogenesis Anti-fibrosis	[29]

#### 5.2.3 SCEV-miRNAs and peripheral vascular age-related diseases

Peripheral artery disease (PAD), pulmonary hypertension (PAH), chronic kidney disease (CKD), and erectile dysfunction are age-related peripheral vascular diseases [86]. An increasing number of studies have focused on the therapeutic effects of SCEV-miRNAs on age-related peripheral vascular diseases (Table 5). The prevalence and incidence of PAD are highly age-related [87]. Beyond atherosclerosis, accelerated vascular aging might form the basis of the pathological manifestations of PAD [88]. MiR-675 is an essential regulator of cellular senescence. Melatonin also attenuates senescence-associated proliferation reductions that antagonize premature CPC senescence via the H19/miR-675/USP10 pathway [89]. MiR-675 participates in the anti-aging pathway of melatonin (MT) in premature cardiac senescence and regulates vascular cell dysfunction by downregulating the TGF-β-1/pSMAD/p21 pathway. Furthermore, aging rats display a down regulation of miR-675. The delivery of miR-675 by SCEVs prevents aging-induced vascular dysfunction in the mouse hind limb. The therapeutic effect of EVs is significantly enhanced by encapsulation in a silk fibroin hydrogel. That study further identified the signaling pathway that mediates aging-induced vascular dysfunction and is the basis for developing an exosome-based therapy to inhibit the aging process [31]. In addition, miR-199b-5p, miR-143-3p, miR-291b, and miR-20b-5p could also be involved in regulating age-related endothelial dysfunction and the prevention of aging. For example, in a mouse model of ischemic limb, miR-199b-5p levels in induced pluripotent stem cell (iPSC)-derived endothelial exosomes were upregulated, inhibiting the Jagged-1-dependent upregulation of VEGFR2 and promoting age-related EC migration, proliferation, and angiogenesis [38]. In addition, exosomes derived from induced vascular progenitor cells (iVPCs) promoted angiogenesis, *in vitro* and *in vivo*, in a rat hind limb ischemia model. This therapeutic effect of iVPC-EXOs is thought to be related to the expression of age-related exosomal miR-143p, miR-20b-5p, and miR-291b [90].

PAH is characterized by dysfunction of the pulmonary artery endothelium and particularly by excessive proliferation. Pulmonary artery aging is an important cause of PAH associated with age-related pulmonary artery dysfunction. Excessive EC proliferation results in pulmonary vascular remodeling and vascular occlusion, eventually causing end-stage irreversible pulmonary hypertension [91, 92]. According to subclinical studies, SCEV-miRNAs enable the recovery of endothelial function to cure age-related PAH [93]. As previously mentioned, both miR-17 and miR-204 regulate aging [94, 95]. One study showed that the intravenous delivery of MSC-EVs could inhibit age-related PAH in a rat model of hypoxic PAH [93]. Sequence analysis revealed downregulation of the miR-17 superfamily and upregulation of miR-204 levels in ECs. This inhibited the STAT signaling pathway, and thus regulated excessive proliferation and ameliorating of age-related PAH [93]. Furthermore, miR-191, overexpressed in age-related PAH, promotes the proliferation and migration of ECs [32]. The downregulation of miR-191 activates bone morphogenetic protein receptor 2 (BMPR2) mutations by preventing BMPR2 degradation, which inhibits exaggerated inflammatory responses, rescues age-related endothelial dysfunction, and reverses PAH [41, 96].

The characterization of CKD includes a progressive vascular disease with systemic inflammation, muscle wasting, and weakness. Multiple mechanisms might be at play in the pathogenesis of vascular aging in CKD. Cell senescence and SASP-related chronic inflammation play essential roles in this disease. In an experimental mouse model of acute ischemia and hyper-perfusion injury, RAPC-derived exosomes were enriched in miR-218, which is highly expressed in aging mice. This miRNA improves renal function by targeting *Robo1* mRNA in ECs and promoting cell migration. Studies have shown the potential of RAPC autologous transplantation for the treatment of vascular age-related diseases [42, 97]. Vascular aging also contributes to erectile dysfunction. ADSC-derived exosomes are rich in pro-angiogenic miRNAs (miR-126, miR-130a, and miR-132) and the anti-fibrotic miRNA family (miR-let7b and miR-let7c), which restore erectile function and reduce cavernous fibrosis [98].

## 6. Perspectives for clinical translation

Theoretically, the chemically synthesized microRNAs are also reasonable to treat vascular age-related diseases. However, the use of chemically-synthesized miRNA mimics or antagonists is limited by low transfection efficacy or rate-limiting miRNA processing. Several limitations may also hamper their translation into the clinical practice, such as off-target effects, immune stimulation [99]. Whereas there are some advantages in using stem cell-derived microRNAs for the treatment of vascular age-related diseases. For example, many types of anti-aging microRNAs such as miR-146a/b, miR-183, miR-335, miR-128, miR-133a, and miR-155 are contained in the stem cell-derived exosomes [15]. In addition, stem cells can facilitate cell-cell communication via the dynamic release of exosomes. The biological and structural features of exosomes make them especially ideal for exogenous miRNA delivery and more suitable for clinical practice and facilitate trafficking of miRNAs into the site of action [31]. SCEV-miRNA as therapy for vascular age-related diseases has tremendous prospects. However, its clinical application has certain limitations. First, the particular mechanism of vascular aging remains unclear, which is restrictive for studies. Most studies on SCEV-miRNA are subclinical and have mainly focused on a specific aging disease, such as cancer, stroke, or osteoarthritis; moreover, they are based on the acute phase of the disease [99]. Second, there are concerns regarding the large-scale production of SCEV-miRNA *in vitro*. SCEVs are heterogeneous with different subgroups and varying cargoes. The expansion, optimal isolation, purification, scaled-up cultivation, efficient release, and subsequent RNA quantification and stability tests of SCEVs should be further explored [100].

Furthermore, the safety of SCEV-miRNA should be assessed carefully before clinical application *in vivo*. Unmodified exosomes accumulate predominantly in the liver, spleen, and lung, with lower levels detected in target issues [93]. Finally, SCEV-miRNA accounts for a small percentage of exosomal cargo. Rather than a one-to-one intervention, it also regulates vascular age-related phenotypes through complex genetic and epigenetic regulatory networks.

**Figure 5.**
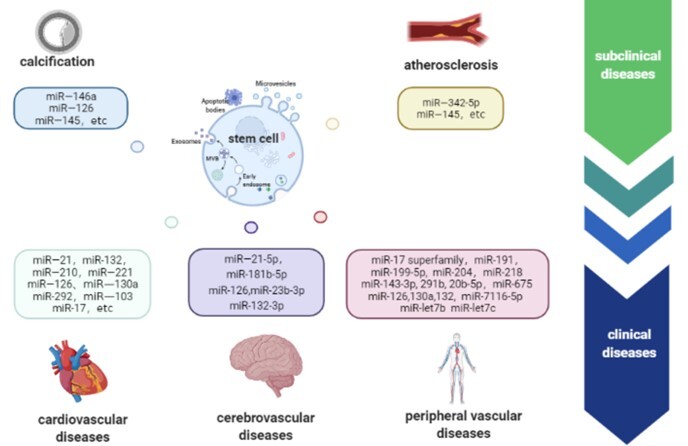
Extracellular microRNAs derived from stem cells (SCEV-miRNAs) in the treatment of subclinical and clinical vascular aging diseases.

In conclusion, the clinical application of SCEV-miRNAs for vascular age-related diseases is promising (Fig. 5). Although the number of studies is increasing, the exact role of SCEVs in the anti-aging mechanism and regenerative effects remains unclear. Further exploration of the specific molecular mechanisms of SCEV-miRNAs and additional data from pre-clinical and clinical studies are required. Several challenges must be overcome to drive SCEV-miRNAs that suppress vascular age-related diseases towards large-scale production and clinical translation.
